# Capnogram plateau micro‑oscillations preceding end‑tidal CO_2_ decline as an early cue to venous air embolism during supratentorial craniotomy: a case report

**DOI:** 10.1186/s40981-025-00841-5

**Published:** 2025-12-11

**Authors:** Ayumu Matsumoto, Noriko Takeno, Michiyoshi Sanuki

**Affiliations:** 1 Department of Anesthesiology, Critical Care and Pain Medicine, NHO Kure Medical Center and Chugoku Cancer Center, 3‑1 Aoyama‑cho, Kure, Hiroshima 737‑0023 Japan; 2 Department of Anesthesiology and Critical Care, Hiroshima University, Hiroshima, Japan

**Keywords:** Venous air embolism, Capnography, End-tidal carbon dioxide, Neurosurgery, Patient safety, Monitoring, Case report

## Abstract

**Background:**

Venous air embolism (VAE) is a potentially fatal complication of craniotomy. When a TEE is not used, attention to waveform-level changes during conventional monitoring is crucial.

**Case presentation:**

A woman in her 70 s underwent supratentorial tumor resection in the supine position with a slight head-up tilt. Approximately 1 h after the craniotomy, gradual decreases in SpO_2_ (98% → 96%), systolic blood pressure (105 → 90 mmHg), and EtCO_2_ (37 → 30 mmHg) were observed. New micro-oscillations appeared on the capnogram plateau, followed by an EtCO_2_ decrease to 22 mm Hg. VAE was suspected. Treatment, including field flooding, head-down positioning, as well as FiO_2_, dobutamine, and rapid fluid supplementation, stabilized her and allowed the uneventful completion of surgery. Arterial blood gas levels improved concurrently with EtCO_2_ recovery.

**Conclusions:**

Careful scrutiny of capnogram morphology, not only numeric EtCO_2_, can expedite the suspicion and timely management of VAE when advanced monitoring is unavailable.

## Background

Venous air embolism (VAE) is a well-recognized complication of neurosurgical procedures. Although traditionally associated with the seated position, air aspiration can occur in various positions because of the hydrostatic gradient between the open venous pathways and the right atrium. Highly sensitive detection methods, such as transesophageal echocardiography (TEE) and precordial Doppler ultrasonography, have applications outside of neurosurgery. However, in frontal lobe craniotomies that require motor-evoked potential (MEP) monitoring, TEE is impractical because of concerns regarding maintaining a sterile surgical field and the risk of coughing or body movements arising from restrictions on neuromuscular blockers. In such cases, standard intraoperative monitoring, particularly capnography, often combined with hemodynamic trends, constitutes the primary practical means of raising suspicion for VAE. Notably, numeric indices, such as EtCO_2_, SpO_2_, and arterial pressure, often lag behind early perfusion changes. Subtle alterations in capnogram waveform morphology (for example, new plateau oscillations or changes in slope) may precede numerical changes in EtCO_2_, potentially offering an earlier window for clinical suspicion and intervention [1–5]. However, the significance of such waveform changes during supratentorial craniotomy in the supine, modestly head-elevated position has received less emphasis, despite ubiquitous use of this position.

Herein, we describe a patient in whom capnogram plateau micro‑oscillations were observed prior to a sudden decline in EtCO_2_, as an early cue to VAE during supratentorial craniotomy.

## Case presentation

A woman in her 70 s with a history of hypertension and transient ischemic attack underwent elective right medial frontal tumor resection. The patient was positioned supine with a head-up tilt of approximately 10°. Tracheal intubation was performed after induction of anesthesia using propofol, remifentanil, and rocuronium. Ventilation was performed using pressure-regulated volume control (PRVC) (tidal volume, 420 mL; positive end-expiratory pressure (PEEP), 3 cm H_2_O; respiratory rate, 12 breaths/min) with a fresh gas flow of 4 L/min. Standard monitoring included electrocardiography (ECG), peripheral oxygen saturation (SpO_2_), invasive arterial pressure, and capnography.

**Fig. 1:**
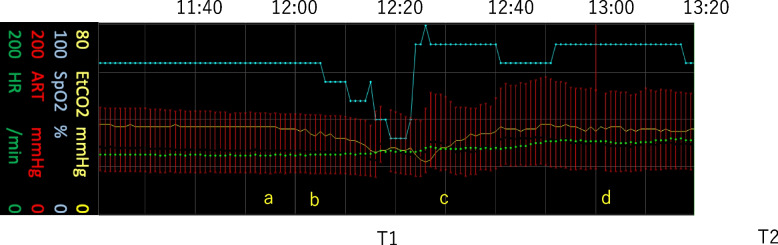
Intraoperative trends around the event. Time-aligned trends showing gradual declines in peripheral oxygen saturation (SpO_2_) (98 → 96%), systolic arterial pressure (105 → 90 mmHg), and end-tidal carbon dioxide (EtCO_2_) (37 → 30 mmHg) over approximately 15 min before the acute EtCO_2_ drop. Symbols a–d in Fig. 1 correspond to a–d in Fig. 2. Representative arterial blood gas (ABG) values are summarized in Table 1

**Fig. 2:**
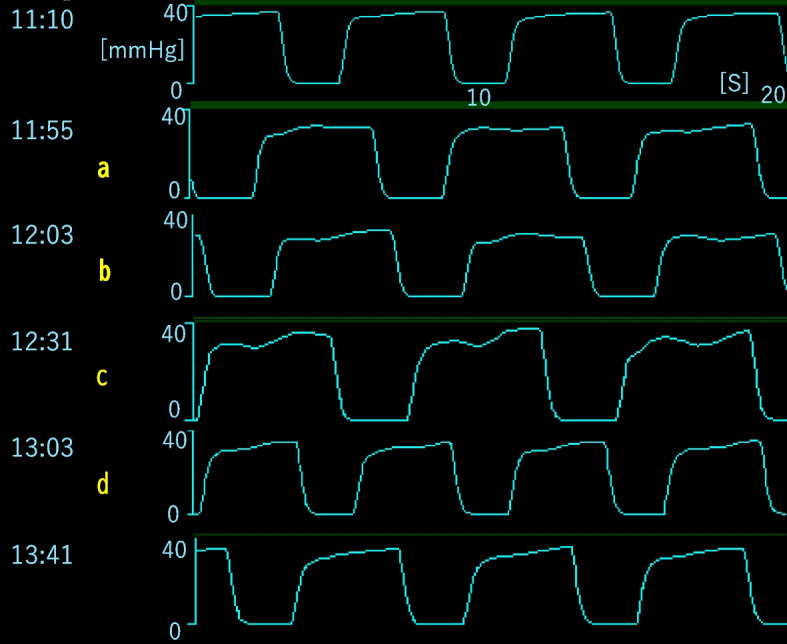
Capnogram plateau micro‑oscillations preceding EtCO_2_ decline. Capnogram segments illustrating newly emerging micro-oscillations on the alveolar plateau that preceded a sharp decrease in EtCO_2_ to a nadir of 22 mmHg. The waveform morphology was qualitatively distinct from the baseline traces (11:10). As EtCO_2_ declined to a nadir of 22 mmHg, the micro-oscillations progressively increased in amplitude (c: 12:31). Following treatment, the oscillations gradually diminished (d: 1 3:03, 13:41), indicating the restoration of more uniform pulmonary perfusion

*Approximately 1 h after dural opening during tumor debulking*, the SpO_2_ gradually decreased from 98 to 96%, systolic blood pressure from 105 to 90 mmHg, and EtCO_2_ from 37 to 30 mmHg over 15 min (Fig. 1a), suggesting evolving hemodynamic and ventilatory compromise. Immediately before a further, abrupt decline in EtCO_2_, newly appearing fine micro-oscillations were noted on the alveolar plateau (Fig. 2a). As EtCO_2_ fell to a nadir of 22 mmHg, these plateau micro-oscillations progressively increased in amplitude (Fig. 2c).

**Table 1:** Representative arterial blood gas and EtCO_2_ levels

Time point	PaO_2_ (mmHg)	PaCO_2_ (mmHg)	P/F	EtCO_2_ (mmHg)	PaCO_2_-EtCO_2_ gradient (mmHg)
During the event (T1)	81	46	203	28	18
After stabilization (T2)	169	43.9	241	36	7.9

EtCO_2_, end-tidal carbon dioxide; P/F, arterial oxygen partial pressure (PaO_2_)/fractional inspired oxygen (FiO_2_); PaCO_2_, partial pressure of carbon dioxide in arterial blood

Values correspond with representative time points referenced in the text and figures. The EtCO_2_ nadir was 22 mmHg. The widened PaCO_2_-EtCO_2_ gradient at T1 (18 mmHg) indicates increased alveolar dead space due to impaired pulmonary perfusion, consistent with VAE. Following treatment, the gradient narrowed to 7.9 mmHg at T2, reflecting restoration of more normal ventilation-perfusion matching

On suspecting VAE, we performed field flooding with saline-soaked gauze, instituted head-down repositioning, increased FiO_2_ to 1.0, initiated dobutamine 5 μg/kg/min, and started rapid crystalloid infusion. Arterial blood gas levels measured at the event (T1) showed a PaO_2_/FiO_2_ ratio of 203, PaCO_2_ of 46 mmHg, and EtCO_2_ of 28 mmHg, indicating an increased intrapulmonary dead space (Table 1). *Within minutes of these interventions, SpO*_*2*_* improved from 96 to 98%, systolic pressure from approximately 90 to 105 mmHg, and EtCO*_*2*_* from a nadir of 22 to 36 mmHg.* Surgery was then resumed. A later arterial blood gas (ABG) (T2) test showed a PaO_2_/FiO_2_ (P/F) ratio of 241, PaCO_2_ of 43.9 mmHg, and EtCO_2_ of 36 mmHg. *She was extubated uneventfully in the operating room and admitted to the intensive care unit for observation. Neurological examination revealed no new deficits; she was alert and oriented, with motor function consistent with her preoperative baseline. Postoperative computed tomography demonstrated expected postoperative changes with no evidence of cerebral infarction, hemorrhage, or pneumocephalus. No recurrent desaturation or hemodynamic instability occurred. She was transferred to the general ward on postoperative day 1 without sequelae related to the intraoperative VAE.*

## Discussion

This case highlights two main practical implications. First, waveform literacy is an important factor. In this patient, new micro-oscillations on the plateau preceded the sharp decline in EtCO_2_ and hemodynamic compromise. This sequence is physiologically plausible. Air entrainment increases alveolar dead space and right ventricular afterload, reducing CO_2_ delivery to the lungs until a threshold is crossed, at which point EtCO_2_ falls steeply. Recognizing qualitative waveform changes can enable earlier suspicion and intervention than relying solely on numerical trends. *Prior reports, mainly in sitting or lateral neurosurgical positions, have linked abnormal capnogram patterns (including plateau alterations and oscillations) with VAE-related EtCO*_*2*_* changes *[4, 5]*.*

*Our case extends these observations in three important ways: First, this case demonstrates that characteristic plateau micro-oscillations can occur during supratentorial surgery with only modest head elevation (10°), a more common position in which VAE risk may be underestimated. Second, waveform changes preceded the pronounced numerical decline in EtCO*_*2*_ *; after a gradual decrease from 37 to 30 mmHg, micro-oscillations appeared before the sharp drop to 22 mmHg, providing an earlier diagnostic window than numerical monitoring alone. This temporal sequence suggests that vigilant waveform assessment may enable intervention before significant hemodynamic deterioration occurs. Third, prompt recognition and management were achieved without TEE or precordial Doppler, emphasizing that standard capnography, when interpreted at the waveform level, remains a practical and effective early-warning tool even when advanced monitors are unavailable or contraindicated (e.g., when MEP monitoring limits neuromuscular blocker use).*

*We observed newly appearing, fine oscillations confined to the alveolar plateau that progressively increased in amplitude before the abrupt fall in EtCO*_*2*_ [4]. *Mechanistically, transient pulsatile obstruction and bubble-induced heterogeneity of pulmonary perfusion could generate flow-linked contour undulations on the plateau, analogous to cardiogenic oscillations described in other capnography contexts. We considered and deemed less likely cardiogenic oscillations, which are typically small, heart-rate–synchronous ripples without concurrent hemodynamic or EtCO*_*2*_* deterioration, as well as circuit artifacts (no changes in ventilator settings, no leak, or sampling-line issues). Patient movement was also unlikely, as there were no changes in neuromuscular management or contemporaneous surgical manipulation. The oscillations intensified in parallel with the EtCO*_*2*_* decline and resolved with VAE-directed measures, supporting pathophysiological plausibility.*

Second, this case underscores that risk is not limited to the seated position; even modest head elevation, as used here, can create a hydrostatic gradient that favors air entrainment via venous channels. Contemporary series and physiological analyses support a position‑agnostic vigilance mindset [2, 3, 6, 7].

The management principles are well established. These include field flooding/water seal, head‑down positioning (left lateral as needed), increasing FiO_2_, hemodynamic support, volume loading, and aspiration via a central venous catheter when available [1]. With respect to ventilatory settings, recent reviews suggest that a higher PEEP does not reliably prevent VAE and may worsen hemodynamics or intracranial physiology, and that individualized titration is prudent [7, 8]. VAE can also occur during repositioning after seated procedures; therefore, vigilance should be continued *even after returning the patient to a horizontal supine position *[9]*. Although our case occurred without major repositioning, this reinforces the need for sustained waveform-level surveillance throughout the procedure and emergence*.

TEE is extremely sensitive for detecting air emboli because it can directly visualize the intracardiac space. It is also useful for evaluating paradoxical air emboli. Recent advances in machine-learning–based approaches for automated VAE detection using TEE image streams suggest that TEE may become an even more powerful complement to intraoperative monitoring in the future [10]. However, several practical considerations arise in supratentorial craniotomy. First, TEE probe insertion and positioning can be challenging, with substantial head rotation for surgical access. Second, neurosurgical procedures often limit neuromuscular blockade to permit MEP monitoring, thereby increasing the risk of patient movement, coughing, or esophageal trauma during probe manipulation. Third, prolonged probe placement during lengthy procedures carries the risk of pressure injury. Fourth, TEE interpretation requires specialized training that may not be universally available. Given these considerations, the decision to use TEE should be individualized according to patient risk factors, surgical requirements, and institutional resources, while ensuring that its use does not delay other time-critical resuscitative measures. When a TEE is not feasible, heightened attention to standard waveform-based monitoring becomes essential.

*In this case, newly appearing and intensifying Capnogram plateau micro-oscillations preceded an EtCO*_*2*_ *fall and signaled evolving VAE during non-sitting craniotomy, suggesting that waveform-level assessment may provide an early warning cue in similar settings. Embedding “waveform-first” checks into VAE algorithms may expedite targeted actions (field flooding, head-down, FiO*_*2*_ *uptitration, and hemodynamic support) when advanced monitors are unavailable.*

## Data Availability

All data supporting the observations are included in this article, and additional de-identified data are available from the corresponding author upon reasonable request.

## Abbreviations

VAE: Venous air embolism
EtCO_2_: End-tidal carbon dioxide
TEE: Transesophageal echocardiography
PEEP: Positive end-expiratory pressure
ICU: Intensive care unit
PRVC: Pressure-regulated volume control
FiO_2_: Fraction of inspired oxygen
SpO_2_: Arterial oxygen saturation
ECG: Electrocardiogram
MEP: Motor-evoked potential
